# A mixed methods protocol to evaluate the effectiveness and acceptability of COVID-19 Community Assessment Hubs

**DOI:** 10.12688/hrbopenres.13217.2

**Published:** 2021-05-12

**Authors:** Sophie Mulcahy Symmons, Robert Fox, Marese Mannion, David Joyce, Aoife De Brún, Liam Glynn, Damien Ryan, Niamh Keane, Eilish McAuliffe

**Affiliations:** 1 Centre for Interdisciplinary Research Education and Innovation in Health Systems (IRIS), School of Nursing, Midwifery and Health Systems, University College Dublin, Dublin, D04 V1W8, Ireland; 2 School of Medicine, University of Limerick, Limerick, V94 T9PX, Ireland; 3 HRB Primary Care Clinical Trials Network Ireland, Galway, Ireland; 4 ALERT, Emergency Department, University Hospital Limerick, Limerick, V94 F858, Ireland; 5 Department of Public health Nursing, Health Service Executive, Dublin, Ireland

**Keywords:** Community Assessment Hubs, COVID-19, coronavirus, patient experience, staff experience, mixed methods

## Abstract

**Background:** Ireland’s health system has been under significant strain due to staff shortages and inadequate capacity. Critical care bed capacity per capita in Ireland is among the lowest in Europe, thus, the coronavirus disease 2019 (COVID-19) pandemic has put additional strain on an over-stretched system. COVID-19 Community Assessment Hubs (CAHs) were established to mitigate unnecessary admission to acute hospitals, and reduce infection spread by supporting COVID-19 positive or suspected positive patients to isolate at home, or in isolation facilities. There is some evidence that similar assessment centres may be a successful triage strategy to reduce burden on hospital and acute care.

**Aim** *: *The aim of this study is to evaluate the impact of COVID-19 Community Assessment Hubs on service delivery in two regions in Ireland during the pandemic.

**Methods:** A mixed-methods approach will be used, incorporating co-design to engage stakeholders and ensure informed data capture and analysis. Online surveys will assess CAH patients’ experiences of access to and quality of care. Clinical patient data from CAHs will be collected and analysed using multinomial logistic regression to check for association with patient demographics and COVID-19 symptoms, and CAH early warning scores and outcomes (Transfer to Emergency Department, Transfer to isolation unit, Sent home with care plan). Semi-structured interviews will be conducted with: patients to elicit an in-depth understanding of experiences and acceptability of attending CAHs; and staff to understand challenges, benefits, and effectiveness of CAHs. Interview data will be analysed using thematic analysis.

**Discussion**: This study will provide valuable insights from both patient and staff perspectives on the operation of CAHs. We will evaluate the effectiveness and acceptability of CAHs and propose areas for improvement of the service. This will contribute to international literature on the use of community assessment centres during infectious disease pandemics.

## Introduction

 The coronavirus disease 2019 (COVID-19) pandemic has created substantial demands on the Irish healthcare system. Irish hospitals operate at near full capacity on a regular basis ( Keegan *et al.*, 2019), and the COVID-19 pandemic has raised concerns whether all COVID-19 patients can receive the care they need. The low ratio of ICU beds to population size compared to other countries is of concern ( Keegan *et al.*, 2019; Rhodes *et al.*, 2012), given late presentation to hospital and subsequent rapid deterioration, resulting in more patients requiring admission to the intensive care unit (ICU). High levels of COVID-19 amongst healthcare staff adds to the existing staff shortages and demand on the system ( Kennelly *et al.*, 2020). Ireland’s low GP to population ratio ( Teljeur *et al.*, 2010) has meant that primary care is experiencing challenges managing the surges in COVID-19 positive or suspected positive patients. The healthcare system in Ireland is not well-equipped to manage an escalating number of people presenting with COVID-19 symptoms, mild or severe.

We now have evidence that the majority of COVID-19 patients suffer with mild to moderate symptoms and can be cared for remotely in self-isolation ( Greenhalgh *et al.*, 2020), however, severe cases must receive timely care. The ability to detect deterioration in suspected and COVID-19 positive patients early and direct them to the appropriate care setting would have a substantial impact on efficient resource utilisation and patient outcomes. Well planned triage strategies in emergency situations can enable more efficient resource utilisation and improve decision-making, particularly important for critical care allocation ( Christian, 2019). Triage can ensure that the most appropriate care is provided in the most appropriate setting.

The World Health Organisation (WHO) recommended expanding screening and referral pathways in community settings to ensure preparedness for the COVID-19 pandemic and provide primary care surge capacity ( World Health Organisation, 2020). Such examples include fever clinics or influenza assessment centres. Assessment centres were set up globally to protect health systems from over-stretching capacity during the COVID-19 pandemic ( Huston *et al.*, 2020). These centres provide specialist services to test, assess, triage, and treat COVID-19 positive or suspected COVID-19 patients in the community rather than in hospitals, but vary in the range of services provided depending on place. Patients receive care plans tailored to their needs and it allows fast-track of severe cases to hospital for more advanced care. Although limited, emerging evidence does suggest that these centres can contribute to reducing the burden on overwhelmed hospitals and acute services ( Hall *et al.*, 2013; John *et al.*, 2020; Wang *et al.*, 2020; Yen *et al.*, 2014), allowing other primary care services to run as normal whilst mitigating the risk of infection.

Evidence from the H1N1 influenza epidemic in 2009 suggests that these centres successfully reduced Emergency Department (ED) volumes and avoided overwhelming hospitals in Canada and Taiwan ( Hall *et al.*, 2013; Yen *et al.*, 2014). In Taiwan, assessment centres, along with other infection control procedures, were suspected to delay the peak of H1N1 pandemic while vaccines were under development ( Yen *et al.*, 2014). Similarly early evidence has shown that fever clinics in China, initially established during the SARS outbreak, were repurposed for the COVID-19 pandemic and reduced infection spread and ED visits ( Wang *et al.*, 2020). In Massachusetts, USA, a community COVID-19 management model was set up, which included telehealth, assessments for testing and advice for self-isolation, and respiratory centre for in-person visits ( John *et al.*, 2020). This community model of care for COVID-19 patients enabled 92% of patients to be managed at home, and resulted in fewer ED visits compared to the national average ( John *et al.*, 2020). In England, primary care assessment services were set up to assess COVID-19 or suspected COVID-19 patients and were primarily led by GPs. However, these services lacked central guidance and many closed after evidence showed that utilisation of hubs was low, with responsibility reverting back to GP practices ( Majeed *et al.*, 2020). A recent paper evaluates the impact of the service in one region in London, which focused on staff acceptability of the service ( Hibberd *et al.*, 2021). Here, approximately half of appointments were dealt with remotely, the rest were either home visits or face to face appointments at the COVID-19 centre, following these appointments less than 10% were referred to hospital. Surveyed staff who worked in or referred to the service felt it reduced infection spread and was a safe way to assess patients. They also reported that this model enabled sharing of “local knowledge” and knowledge of COVID-19. This increased confidence of GPs seeing COVID-19 patients, as well as positive sentiment towards the collaborative environment. This sets precedence for more evaluations of these types of services to understand their role during health emergencies and future utility.

**Figure 1.**
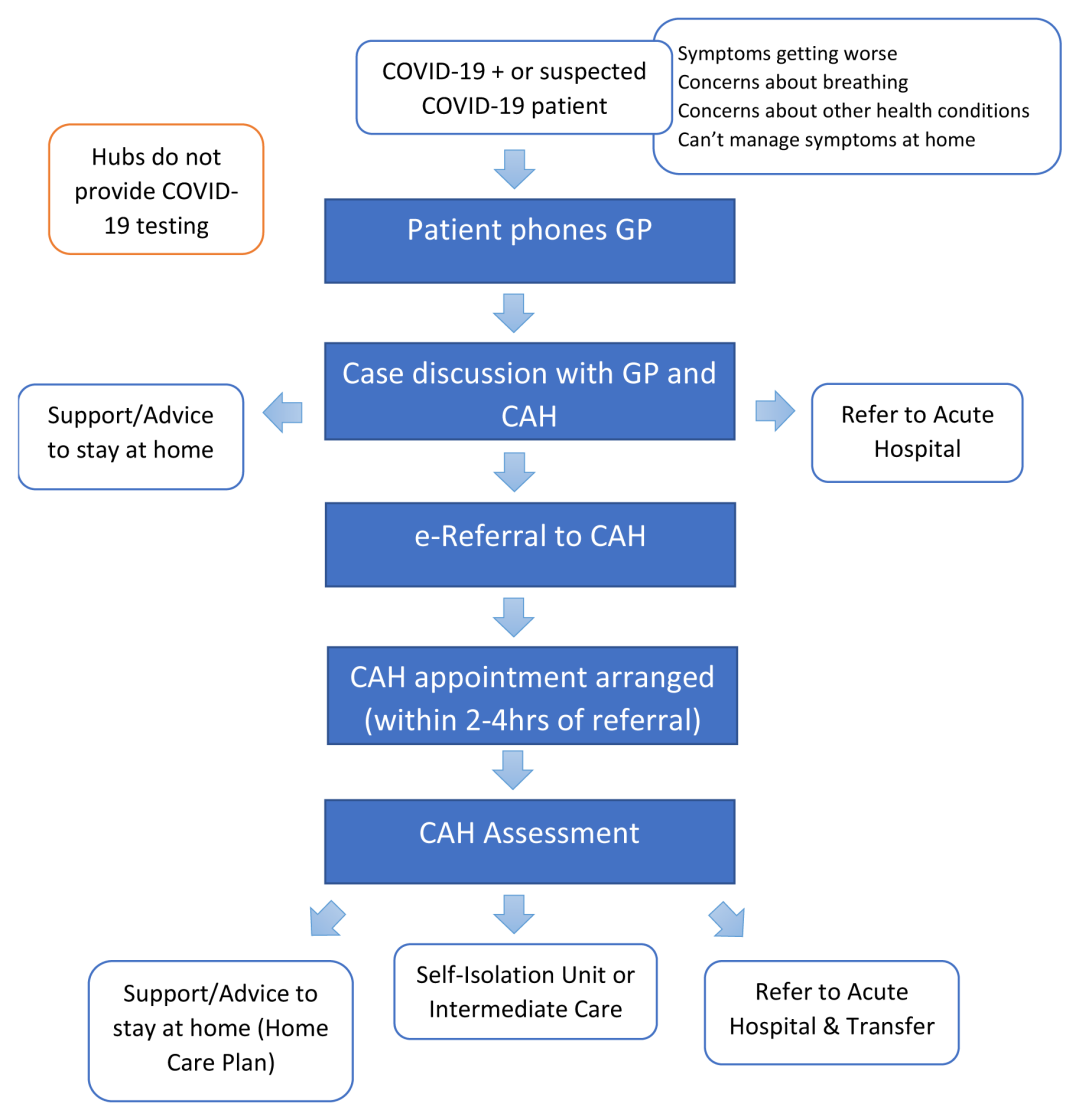
Community Assessment Hub Pathway. Patient referral and assessment pathway. Adapted from ( Nolan & Ní Bhriain, 2020)].

Following the WHO guidance, the Irish health service established COVID-19 Community Assessment Hubs (CAHs) in April 2020 to provide timely specialised services for COVID-19 positive or suspected patients in need of care ( Nolan & Ní Bhriain, 2020). These hubs were unique to influenza assessment centres and more similar to the service model in England; they required direct referral from a GP and did not provide testing or treatment. Suspected or positive COVID-19 patients were referred to a CAH by their GP if their symptoms were getting worse, they had concerns about breathing, other health conditions or could not manage symptoms at home ( Health Service Executive, 2020). These hubs were staffed voluntarily by GPs, but registrars and primary care staff were also redeployed to CAHs ( Bury *et al.*, 2020). Staff deemed high risk of severe COVID-19 (over 60 years, have pre-existing health condition, pregnant) were not asked to work at these hubs. The hubs consisted of a multi-disciplinary team of GPs, nurses, physiotherapists, and administrative staff. The aim of the hubs was to prevent patients from overburdening the hospital system by providing timely community-based care to COVID-19 positive or suspected positive patients, avoid unnecessary attendance to acute hospital, and maximise the number of patients who can self-isolate at home ( Nolan & Ní Bhriain, 2020). GPs referred deteriorating patients (i.e. patients who could not manage symptoms at home) to these hubs for assessment where a decision to was made to refer to ED, refer to a self-isolation unit or self-isolate at home (see Figure 1 for CAH pathway). Patients were triaged at two stages; firstly, when the referring GP discussed the case with the CAH, and secondly when CAH assessment was complete. The service aimed to take referrals and provide appointments within 2–4 hours. CAHs were staffed for 12 hours a day, 7 days a week, but this reduced to reflect demand over time. The benefits of this approach include reducing infection risks associated with in-person GP attendances and allowing GP services to operate normally, reduction in ED attendance and hospital bed occupancy, and reassurance for the patient and the GP after clear care plans are provided by CAHs. Without this triage strategy, Ireland’s GP and acute care services may have been less capable of managing patients and infection spread, potentially resulting in the health system being unable to manage the volume of COVID-19 patients. To our knowledge, there has been one study to investigate CAHs in Ireland that focused on the infection control training that staff received and voluntary uptake to work shifts in these hubs ( Bury *et al.*, 2020). This study found that compliance with infection control procedures was high, and staff had positive experiences working in the hubs ( Bury *et al.*, 2020).

To date, there is a dearth of research evaluating these centres. In addition, there is very little published on patient experiences of this type of care ( Fitzgerald, 2020). We hope to address this gap by exploring the impact of CAHs on service delivery in two regions in Ireland during the time they were open in 2020 and 2021, with focus on the first peak of the pandemic April–June 2020. All hubs in these regions have since closed at the end of March 2021. The aim of this retrospective study is to assess the effectiveness and acceptability of CAHs and identify how the service might be improved or adapted for future waves of COVID-19 and other public health emergencies. This will be achieved by: assessing patient predictors of CAH acceptability; exploring patient experience of CAHs; exploring staff experiences of the acceptability and challenges of CAHs; investigating the predictors for CAH outcomes using CAH patient data. Patient and staff perspectives will be gathered using online surveys and interviews from CAHs in two regions in Ireland that were operational during the first peak of the COVID-19 pandemic up until their closure. Patient outcomes will be analysed using clinical patient data from CAHs in two regions in Ireland. Our mixed-methods approach will provide comprehensive findings to inform whether CAHs were acceptable and effective during the pandemic.

## Methods

### Research design

This mixed-methods retrospective study will adopt an iterative approach, utilising the principles of co-design to engage stakeholders to ensure the optimum study design, efficient data capture, informed analysis, and interpretation of findings as they emerge, and timely and continuous dissemination, to inform the future utility of CAHs now or in future public health emergencies. 

There will be several components to this retrospective study: (1) cross-sectional study of patient experience using interviews and surveys, (2) cross-sectional study of healthcare staff experience using interviews, and (3) analysis of existing patient data from CAHs to predict patient outcomes. Data for each component of the study will pertain to the time from the opening of the CAHs in April 2020 to their closure for both regions in Ireland.

### Methods and analysis

***Cross-sectional study of patient experiences.*** Data will be gathered from a cohort of patients, assessed in the CAHs. All patients from April–June 2020 in Region A and 100 patients assessed in January 2021 in Region B. The dates of data collection for Region B differ to Region A to improve recall and response rate. This will be done via online surveys and interviews to understand their experience using the CAH service. Participants will be recruited via gatekeepers at CAHs in each region. Discharged patients will receive a letter of invitation with a link and QR code to an online survey which also contains the Participant Information Sheet and Consent Form (see extended data ( Mulcahy Symmons *et al.*, 2021)). Through this survey, patients also have the option of providing their contact details to the researchers if they wish to participate in a telephone interview. Additionally, 10 patients in Region B, assessed in January 2021, who were not contacted about the survey will be sent an invitation to take part in the interview only. This is to boost numbers to take part in an interview. These participants will then be contacted by a member of the research team to arrange a suitable time for interview, once 7 days have passed since signing the consent form. All fully consented patients who have been discharged from CAHs are eligible for inclusion in the study, those who can do not have capacity to consent, decline to take part, or are under 18 years will be excluded.

In the survey, participants will be asked to indicate their age range, gender, ethnicity, educational level, COVID-19 symptoms, underlying conditions, and their experience with the CAH service (including information received, access to care, quality of care (using the Patient-Professional Interaction Questionnaire (PPIQ) scale ( Casu *et al.*, 2019)),). Survey data will be quantitatively analysed to determine predictors of overall acceptance of the CAHs. To determine the predictors of patient acceptability of CAHs, multiple regression analysis will be performed using patient quality of care, access and information received, patient demographics (age, gender, ethnicity, and education), COVID-19 symptoms, and access to care as predictor variables.

Approximately 10 follow-up semi-structured interviews will be conducted with patients (on a voluntary basis), 5 from each region, who indicate consent to participate to explore their experience and acceptability of receiving care in the CAHs in detail (see extended data ( Mulcahy Symmons *et al.*, 2021)). Patients will be convenience sampled to ensure rapid collection of data. Thematic analysis of interview transcripts will be conducted to explore in detail perceptions of the CAHs and identify common themes ( Braun & Clarke, 2006). Themes will be drawn out inductively by one researcher and reviewed and discussed with the research team. Interviews will be coded using NVivo 12 software ( QSR International Pty Ltd, 2020). A second researcher will independently code a subset of transcripts to ensure the quality of the research.

***Cross-sectional study of healthcare staff experiences.*** Any member of staff who worked at the CAHs for at least one week during the time the CAHs were open, between April and June in Region A and April 2020–March 2021 in Region B, is eligible to participate in the study. The Participant Information Sheet and Consent Form (see extended data ( Mulcahy Symmons *et al.*, 2021)) for the interview will be emailed to staff via gatekeepers. Staff who consent will then be contacted by the research team to arrange a time for interview 7 days after informed consent is received. Staff who have consent to participate will be selected purposively for a representative sample of the multi-disciplinary teams working at the CAHs.

An interview guide will be co-designed to investigate CAH staff experiences working in CAHs (see extended data ( Mulcahy Symmons *et al.*, 2021)). Semi-structured interviews with approximately 20 staff in total, 15 in Region A and 5 in Region B, will be conducted on the benefits and challenges of CAHs, including changes in clinical practice, communication with and management of patients, and teamwork. Thematic analysis of interview transcripts will be conducted to identify common themes of the challenges and benefits and acceptability of working in CAHs. Initial themes will be identified inductively by one researcher and reviewed with the research team. Interviews will be coded using NVivo 12. Themes will be compared between data in region A and in Region B for any differences.

***Analysis of existing patient data from CAHs.*** Anonymised data on patient symptoms, early warning scores, deterioration, and admissions will be extracted from the CAHs for the period April to June 2020 in both regions. Data will be securely transferred to the research team for quantitative analysis following anonymisation by CAH clinic staff.

Descriptive statistical analysis will be used to provide the sample characteristics. A multinomial logistic regression will be used to determine the association between patient demographics, presumed or COVID-19 positivity, and COVID-19 symptoms, and early warning scores and patient outcomes (transfer to ED, transfer to isolation unit, sent home with care plan). This will allow for determination of the specific variables associated with the different outcomes. 

### Ethics

Ethical approval has been granted by the COVID-19 National Research Ethics Committee (Ref: 20-NREC-COV-093). In accordance with data protection regulations and the data sharing agreement with the partner organisations, patients’ clinical data will be anonymised by on-site healthcare staff and encrypted before being transferred securely to the research team. No identifiable data will be included in the final consolidated dataset. Information sheets will be provided to all prospective interview participants and informed consent will be obtained online for the semi-structured interviews with staff and from patients completing the survey. Participants will be advised that they are free to refuse to answer any questions, are free to withdraw at any time without question or reason and are free to take a break during the interview if required. This information will be clearly stated in the information sheets and verbally confirmed prior to beginning any interview. Identifiable information from interviews will be removed from transcripts.

### Dissemination of information

Results will be disseminated via regular briefings and updates, publication in peer-reviewed journals, national and international conferences where possible, and to relevant stakeholders and interest groups using public forums and social media. Participants who are interested in the study's findings will be invited to make this known to the researchers and a summary of results will be sent to these participants.

### Study status

This study is ongoing. Data collection for all components of the study is completed and analysis is underway.

## Discussion

The overarching aim will be to evaluate CAHs in Ireland and determine their effectiveness using a mixed-methods approach. Quantitative and qualitative data on patient and staff experience will provide a dual lens to holistically capture CAHs in operation. This will also provide insight on patients preferred care pathways and staff’s perceptions of the benefits and challenges of delivering care in this way. We will be able to assess how well the multi-disciplinary teams at the hubs worked together and understand where there is room for improvement at these unusual settings. Clinical data will ensure comprehensive analysis of the effects of CAHs on patient outcomes. We will be able to determine whether CAHs achieved their goal of providing timely community-based care to patients, optimise patient outcomes and minimise unnecessary use of acute hospital capacity by maximising the number of patients self-isolating at home. These findings will provide valuable information on the benefits, limitations, and areas of improvement of CAHs. This will be beneficial to all stakeholders and inform decision-making on the implementation and operation of these or similar hubs in the future. These findings may also aid potential repurposing or integration of other COVID-19 services, e.g., rehabilitation and testing, with CAHs to provide improved care of COVID-19 patients.

The findings from this research will have international relevance as it will contribute to improvement of assessment services during the COVID-19 pandemic, and for future public health emergencies, where assessment centres in the community may need to be set up to address need and mitigate hospital visits. Rapid release of findings is intended for this work to aid service improvement of primary care triaging for COVID-19 patients in a time-sensitive manner. It is essential that health systems adapt to the rapidly changing situation caused by the pandemic. As such, this research should inform service development.

There are several hurdles to overcome to conduct research during a pandemic. One challenge to implementing co-design principles is co-ordinating with busy healthcare collaborators, this will be addressed via flexible remote meeting arrangements and sustained open dialogue across sites. In order to reduce the burden of work on our collaborators at Region B, only a sample of 100 patients will be contacted to participate in the survey as the workload to send out postal invitations is high. Another challenge is recruitment of participants to our study, mainly that patients can only be contacted via post. Therefore, only a link to the survey in a letter invitation can be sent, rather than more conveniently via email. The impact of this limitation will be mitigated through emphasis of the value of the research and the low time commitment to complete the brief online surveys and interviews. Convenience sampling for the qualitative interviews of patients will be used to ensure follow-up interviews can be conducted rapidly.

## Data availability

### Underlying data

No data are associated with this article.

### Extended data

Zenodo: A mixed methods protocol to evaluate the effectiveness and acceptability of COVID-19 Community Assessment Hubs. http://doi.org/10.5281/zenodo.4476556 ( Mulcahy Symmons *et al.*, 2021)

This project contains the following extended data:

○ Staff Interview Guide.docx (Interview guide for staff)
○ SFI20-0221 Consent Form (Staff).docx (Consent form for staff)
○ SFI20-0221 PIL Staff.docx (Participant Information Sheet for staff)
○ Community_Assessment_Hub_patient_survey with consent.docx (Survey for patients (with consent))
○ SFI20-0221 PIL Patients.docx (Participant Information Sheet for patients)
○ Patient Interview Guide.docx (Interview guide for patients)

Data are available under the terms of the Creative Commons Attribution 4.0 International license (CC-BY 4.0).
