# Supplementary material for: A mixed methods protocol to evaluate the effectiveness and acceptability of COVID-19 Community Assessment Hubs
Source: HRB Open Res. 2021 May 12;4:16. Originally published 2021 Feb 9. [Version 2] doi: 10.12688/hrbopenres.13217.2 (PMC8136252; doi:10.12688/hrbopenres.13217.2)
Supplement: Peer review history

# Peer review: Reviewer response for version 2

**DOI:** 10.21956/hrbopenres.14482.r29411

**Published:** 2021-05-19

**Authors:** Gerard Fitzgerald

**Affiliations:** 1 School of Public Health and Social Work, Queensland University of Technology, Kelvin Grove, Qld, Australia

Thank you for asking me to review this article. The authors have responded well to the comment made previously and so the paper is worthy of indexing.

Is the study design appropriate for the research question?

Yes

Is the rationale for, and objectives of, the study clearly described?

Yes

Are sufficient details of the methods provided to allow replication by others?

Yes

Are the datasets clearly presented in a useable and accessible format?

Yes

Reviewer Expertise:

NA

I confirm that I have read this submission and believe that I have an appropriate level of expertise to confirm that it is of an acceptable scientific standard.

# Peer review: Reviewer response for version 2

**DOI:** 10.21956/hrbopenres.14482.r29448

**Published:** 2021-05-14

**Authors:** Trish Greenhalgh

**Affiliations:** 1 Nuffield Department of Primary Care Health Sciences, University of Oxford, Oxford, UK

This is a retrospective protocol on a study for which the definitive paper is likely imminent. It's already been through one iteration of peer review. I won't repeat either what's in the paper or what the previous reviewer said. 

 It's short, clear and relatively comprehensive. I only have one tiny quibble which is I wouldn't cite Greenhalgh *et al *2020 to support the statement "we now have evidence that the majority of covid-19 patients suffer with mild to moderate symptoms and can be cared for remotely in self-isolation". That reference was penned in extreme haste in early March 2020 when we had almost no evidence on any aspect of the disease. We took an educated guess!  I think there is now pretty good evidence (from other studies, sorry I don't have them at my fingertips but they'd be easily hunted down I think) to support the statement, though it should be qualified with the caveat that SOME patients are extremely sick or on a downward trajectory and that safe management in the community is all about ensuring that we can reliably weed out the ones who need escalation of care.  

 The other thing I'd have found useful (but not a requirement, just a suggestion) is more information about healthcare in Ireland. Maybe that's coming in the main paper? 

 I look forward to seeing the results of this interesting study.

Is the study design appropriate for the research question?

Yes

Is the rationale for, and objectives of, the study clearly described?

Yes

Are sufficient details of the methods provided to allow replication by others?

Yes

Are the datasets clearly presented in a useable and accessible format?

Not applicable

Reviewer Expertise:

GP academic

I confirm that I have read this submission and believe that I have an appropriate level of expertise to confirm that it is of an acceptable scientific standard.

# Peer review: Reviewer response for version 1

**DOI:** 10.21956/hrbopenres.14370.r28883

**Published:** 2021-02-19

**Authors:** Gerard Fitzgerald

**Affiliations:** 1 School of Public Health and Social Work, Queensland University of Technology, Kelvin Grove, Qld, Australia

This article outlines a research protocol to evaluate Community Assessment Hubs for COVID 19. These hubs appear to differ from Fever Clinics and testing centers in that they provide specialist assessment but also require GP referral and are not open directly to the public. Thus they act as a substitute for hospital level assessment and are intended to avoid hospital congestion and ED demand. 

 Community assessment hubs have been part of the mixture of health system strategies used across the world to help manage the COVID 19 pandemic. As such they would benefit from a well structured evaluation to determine their role and benefit in reducing hospital demand and achieving community satisfaction.

 The proposal identifies a well structured mixed methods evaluation using both quantitative and qualitative approaches. Perhaps the only additional source of data may be to examine contemporaneous hospital admission and ED attendance data.

 What is less clear is whether this is intended as a retrospective or prospective study and when the study did or shall start. This is evidenced in the paragraph which describes the hub in which the words "..would be established..", "...were staffed..." and ..'"consists of.." are used demonstrating various tenses. It would be beneficial to make this clear to the reader. Publishing a protocol for an extant study is fine. It just needs to be made clear. This is particularly relevant in that the number of cases in Ireland has declined rapidly since  peaks in early January and with the roll out of vaccines, it is hopeful that further widespread transmission may be avoidable. Therefore the ongoing need for community hubs may be lessened and their evaluation in this context less useful.

 My recommendation is that the authors make the starting date for the study explicit and ensure consistency in tense is used throughout the paper.

Is the study design appropriate for the research question?

Yes

Is the rationale for, and objectives of, the study clearly described?

Yes

Are sufficient details of the methods provided to allow replication by others?

Yes

Are the datasets clearly presented in a useable and accessible format?

Yes

Reviewer Expertise:

Emergency health systems

I confirm that I have read this submission and believe that I have an appropriate level of expertise to confirm that it is of an acceptable scientific standard, however I have significant reservations, as outlined above.

# Response

**Published:** 2021-04-27

**Authors:** Sophie Sophie

**Affiliations:** University College Dublin, Ireland

Dear Professor Fitzgerald,

 We thank you for taking the time to review our protocol “A mixed methods protocol to evaluate the effectiveness and acceptability of COVID-19 Community Assessment Hubs”. We have considered your feedback and have revised the manuscript to enhance clarity of the protocol and the timeline of this retrospective study.

 In response to your comment, “ ***My recommendation is that the authors make the starting date for the study explicit and ensure consistency in tense is used throughout the paper***”; Revisions have been made to ensure tense consistency in the 5 ^th^ paragraph of the Introduction section: “ *Following the WHO guidance, the Irish health service established COVID-19 Community Assessment Hubs (CAHs)  in April 2020 to provide timely specialised services for COVID-19 positive or suspected patients in need of care ( Nolan & Ní Bhriain, 2020). These hubs were unique to influenza assessment centres and more similar to the service model in England; they required direct referral from a GP and did not provide testing or treatment. Suspected or positive COVID-19 patients were referred to a CAH by their GP if their symptoms were getting worse, they had concerns about breathing, other health conditions or could not manage symptoms at home ( Health Service Executive, 2020). These hubs were staffed voluntarily by GPs, but registrars and primary care staff were also redeployed to CAHs*”.

 In the last paragraph of the Introduction section the time period for which data was extracted and analysed was stated in the following sentences: “ *We hope to address this gap by exploring the impact of CAHs on service delivery in two regions in Ireland during the time they were open in 2020 and 2021, but focusing on the first peak of the pandemic April-June 2020. All hubs in these regions have since closed at the end of March 2021. The aim of this retrospective study is to assess the effectiveness and acceptability of CAHs and identify how the service might be improved or adapted for future waves of COVID-19 and other public health emergencies*”.

 Clarification of the dates of data collection was also made in the Methods section, as exemplified here: “ *Data will be gathered for each component of the study from the opening of the CAHs in April 2020 to their closure for both regions in Ireland.*” Data collection and dates for collection in some components of the study differ for each region, and this has been stated clearly and justified: “ *Data will be gathered from a cohort of patients, assessed in the CAHs, all patients from April-June 2020 in Region A and 100 patients assessed in January 2021 in Region B. The dates of data collection for Region B differ to Region A to improve recall and response rate.”* Collection of data is near completion and is stated in the Study Status section.

 In response to your suggestion “ ***Perhaps the only additional source of data may be to examine contemporaneous hospital admission and ED attendance data***”; we agree that this would be a valuable addition to the study. However, as this data is not captured in the hubs, and the research team has permission to access the hub data in a fully anonymised format only, it is not possible to match data for analysis of the complete patient pathway. We are collaborating with clinical colleagues in the hospital setting with the aim of generating summary statistics on ED attendance and admission of COVID-19 patients, so may be in a position to make some inferences about the impact of the hubs on ED activity.

 Since this protocol was first published on HRB Open, we have established a collaboration with another Community Assessment Hub in another region in Ireland. We plan to replicate this study in this region in Ireland. The Methods section has been updated to reflect this. We look forward to having enhanced ability to evaluate this service and strengthen the findings of the study. Amendments have been made to the protocol stating this change, as seen above. The manuscript has been reviewed for consistency to reflect this addition to the study.

 Kind Regards,

 Sophie Mulcahy Symmons
